# Using the Baidu index to predict trends in the incidence of tuberculosis in Jiangsu Province, China

**DOI:** 10.3389/fpubh.2023.1203628

**Published:** 2023-07-18

**Authors:** Yue Wang, Haitao Zhou, Li Zheng, Min Li, Bin Hu

**Affiliations:** School of Public Health, Xuzhou Medical University, Xuzhou, Jiangsu, China

**Keywords:** tuberculosis, Baidu index, prediction, multiple linear regression, timely

## Abstract

**Objective:**

To analyze the time series in the correlation between search terms related to tuberculosis (TB) and actual incidence data in China. To screen out the “leading” terms and construct a timely and efficient TB prediction model that can predict the next wave of TB epidemic trend in advance.

**Methods:**

Monthly incidence data of tuberculosis in Jiangsu Province, China, were collected from January 2011 to December 2020. A scoping approach was used to identify TB search terms around common TB terms, prevention, symptoms and treatment. Search terms for Jiangsu Province, China, from January 2011 to December 2020 were collected from the Baidu index database.^1^ Correlation coefficients between search terms and actual incidence were calculated using Python 3.6 software. The multiple linear regression model was constructed using SPSS 26.0 software, which also calculated the goodness of fit and prediction error of the model predictions.

**Results:**

A total of 16 keywords with correlation coefficients greater than 0.6 were screened, of which 11 were the leading terms. The R^2^ of the prediction model was 0.67 and the MAPE was 10.23%.

**Conclusion:**

The TB prediction model based on Baidu Index data was able to predict the next wave of TB epidemic trends and intensity 2 months in advance. This forecasting model is currently only available for Jiangsu Province.

## Background

Tuberculosis (TB) is an infectious disease of the lung caused by *Mycobacterium tuberculosis*, which is mainly transmitted through the respiratory tract and poses a serious threat to human life and health. It’s also the second leading cause of death from infectious diseases. According to the WHO Global Tuberculosis Report 2022, 10.6 million new cases of tuberculosis were reported worldwide in 2021. This represents a 3.6% increase in morbidity compared with 2020. TB deaths increased for the second year in a row since 2019, reversing the declining trend in TB deaths over the past decade. The global TB epidemic is more severe than before.

**Figure 1:**
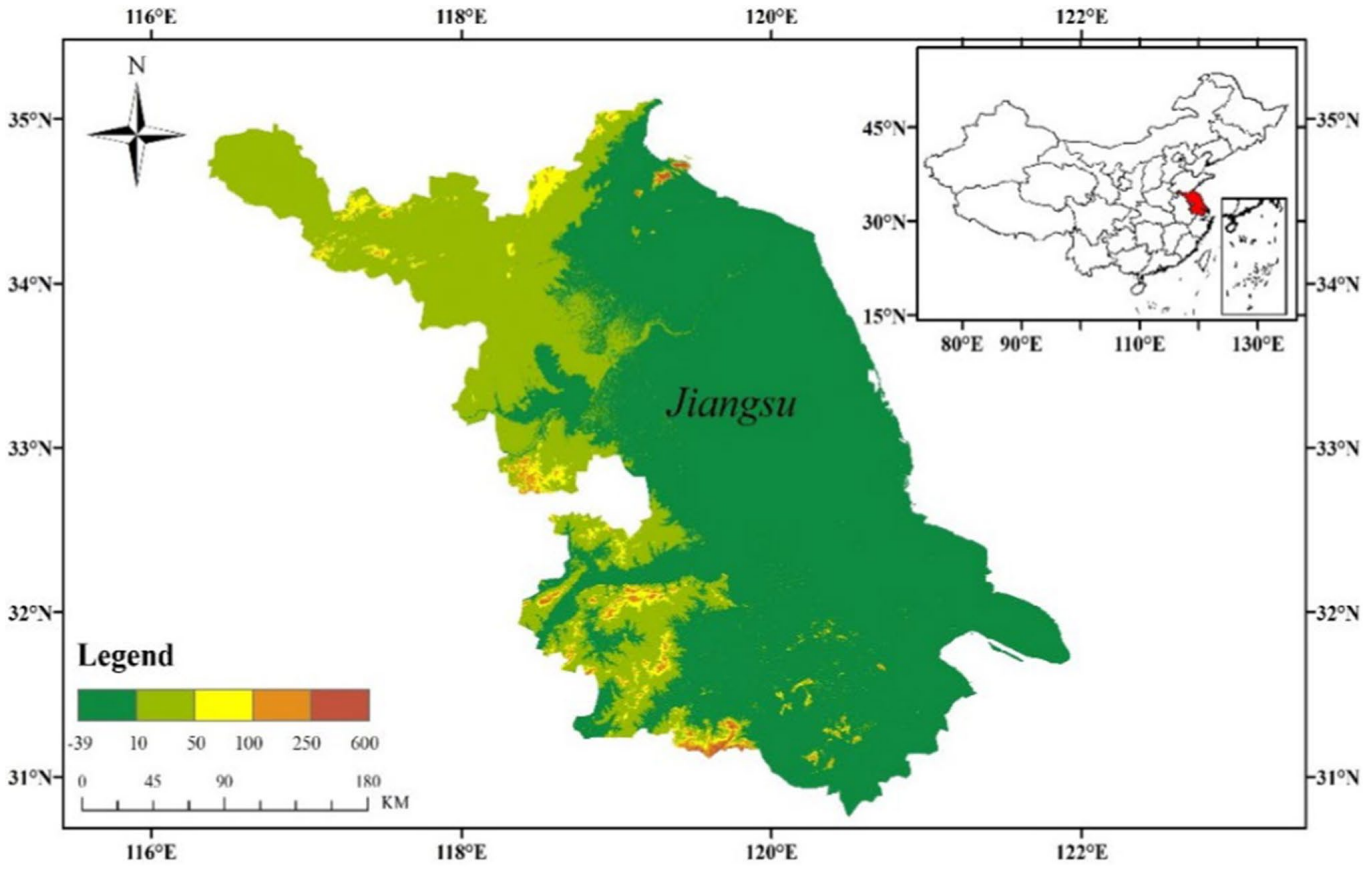
Geographical location of Jiangsu Province.

The incidence of Chinese tuberculosis was the third highest among countries with a high burden of tuberculosis. In China, the mortality rate of tuberculosis was the second highest among the statutory reporting of infectious diseases. The prevalence of tuberculosis was still far from the strategic goal of “Ending TB by 2035.” The early prevention and the timely model for predicting new TB outbreaks can propose early warning of TB outbreaks and monitoring of symptoms. Therefore, effective control of the prevalence and development of TB can minimize the impact on people’s lives and health. Jiangsu Province is located on the east coast of mainland China (Figure 1), spanning 30°45′–35°08′N latitude and 116°21′–121°56′E longitude, with a total area of 107,200 square kilometers. The rate of Internet penetration in Jiangsu Province is 61.5%. The Internet penetration rate of southern regions have reached over 65%, exceeding the national average. The larger sample size of the Internet search data can effectively reduce the data bias caused by insufficient data volume.

Internet query data has been widely used as a new source of data related to early warning and prediction of infectious diseases. Ginsberg et al. (1) used Google to build an influenza prediction model by automatically acquiring search terms. It’s predicted results were 1–2 weeks earlier than traditional CDC surveillance. Li et al. (2) used Twitter data to predict influenza epidemic trends with strong real-time performance. Althouse et al. (3) used Google search engine to monitor dengue-related search terms and built two linear regression models respectively, which was confirmed good correlation between model predicted values and actual surveillance data. In China, Li et al. (4) used Google search engine data and achieved good prediction results through cross-validation analysis. There have been lots of related studies on infectious disease prediction and early warning based on search data at home and abroad. To summarize the above research, we can find that most of the infectious disease surveillance and early warning studies based on Internet data were focused on infectious diseases such as influenza, dengue fever and AIDS (5–8). Meanwhile, Milinovich Gabriel (9) showed that prediction models using Internet data performed better in infectious diseases transmitted through the respiratory tract. But there are few studies on prediction of tuberculosis based on Internet data in China. This is the first time such an Internet search term based early warning surveillance system for TB has been developed. Xue Gong showed that the spatial distribution of Baidu index in China was higher in the eastern region than other region (10). As a result, Jiangsu Province has a large data base, to a certain extent, so it is able to reduce error bias.

According to the 50th Statistical Report on the Development of the Internet in China released by the China Internet Network Information Center, as of June 2022, the Internet penetration rate reached 74.4% and the size of Internet users was 1.051 billion, of which the size of search engine users reached 770 million, accounting for 77.8% of all Internet users. In China, Baidu has become the mainstream search engine. Its market coverage has been accounted for 89.1%. Baidu Index is a China-specific version of Google Trends launched in 2006 (10). Its functions are broadly similar to the Google Trends. Since 2010, when Google Search ceased its services in mainland China, Baidu Index has become the most popular search analysis tool in China (11). Web search data can directly or indirectly reflect the behavior and psychology of Internet users. Some studies on socio-economic activities have attempted to dissect the connotative relationship between search data and the predicted objects. With the rapid development of the Internet and information technology, susceptible people tend to choose to “seek medical consultation” on the Internet (12). So, the search term index covers a large number of early latency and health behavior search information of susceptible people. There are some shortcomings in the existing infectious disease surveillance system (13, 14). Firstly, the traditional infectious disease surveillance and early warning system has a single source of data, which comes from clinical incidence, laboratory surveillance data provided by medical institutions, CDC and sentinel hospitals. Secondly, the acquisition of data was aggregated by departments at all levels after reporting, leading to a relative lag in the early warning gateway and a lack of certain timeliness (15). While the Internet monitoring system avoids the cascading design of traditional monitoring model (16). This paper explains the association between search data and case numbers in terms of individual health status, health information needs and online health information seeking behavior. Whether they are susceptible, latent or infected, people with symptoms of TB will have a need for health information. Baidu, as a common search engine, has become the first choice for searching information, so the Baidu index contains a large number of health information search behaviors. In addition, network search data has the advantages of large sample size, rapid response and ease of access, allowing data to be obtained and predictions to be made in the early symptom period.

## Methods

### Correlation analysis

Correlation analysis is a statistical method for studying the correlation between two and more random variables that are at equal levels. In this study, Pearson’s correlation coefficient was used to describe the correlation between TB data and relevant search terms. In Eq. 1, *X_i_* means the Baidu index of the search term, *Y_i_* is the incidence of TB. The value of *r* is in the range of [−1,1]. The larger the |*r*| means the higher the correlation between the BDI and actual incidence. The initial screening criteria of this study is |*r*| ≥ 0.5, which means the moderate or higher correlation.


(1) $$r=\frac{\overset{n}{\underset{i=1}{\sum }}\left(X_{i}-\bar{X}\right)\left(Y_{i}-\bar{Y}\right)}{\sqrt{\overset{n}{\underset{i=1}{\sum }}{\left(X_{i}-\bar{X}\right)}^{2}\overset{n}{\underset{i=1}{\sum }}{\left(Y_{i}-\bar{Y}\right)}^{2}}}$$

### Correlation time series change characteristics

Time series correlation analysis is the calculation of the correlation coefficient between the time series of the alternative and benchmark indicators after shifting the time units. The calculation formula is given in Eq. 2.


(2) $$r_{d}=\frac{\overset{n}{\underset{i=1}{\sum }}\left(X_{i-d}-\bar{X}\right)\left(Y_{i}-\bar{Y}\right)}{\sqrt{\overset{n}{\underset{i=1}{\sum }}{\left(X_{i-d}-\bar{X}\right)}^{2}\overset{n}{\underset{i=1}{\sum }}{\left(Y_{i}-\bar{Y}\right)}^{2}}}$$

In the Eq. 2, *d* is the lead time, *i* is the reference time, *r_d_* is the time difference correlation coefficient. If *r_d_* is negative, it is the “leading feature,” *r_d_* is “0” and “positive” means “synchronous” and “lagging” feature, respectively. This study used the time sequence change feature to filter out the search terms with “leading” feature.

### Multiple linear regression forecasting

Multiple linear regression is used to analyze the linear relationship between a single dependent variable and multiple independent variables. Based on tolerance and variance inflation factors to determine the multiple covariance between the dependent and independent variables. See Eq. 3 for expression.


(3) $$y=\beta_{0}+\beta_{1}x_{1}+\beta_{2}x_{2}+\beta_{3}x_{3}+\ldots +\beta_{k}x_{k}+\varepsilon$$

In the Eq. 3, y is the number of predicted incidences of tuberculosis, $\beta_{0},\beta_{1},\beta_{2},\ldots ,\beta_{k}$ is the model parameter, $x_{1},x_{2},x_{3},\ldots ,x_{k}$ is the Baidu index of the search term, and ε is the error term that represents the effect of random factors. The study involved 11 variables, so used stepwise regression to avoid overfitting the prediction model.

## Results

### Prevalence profile

**Figure 2:**
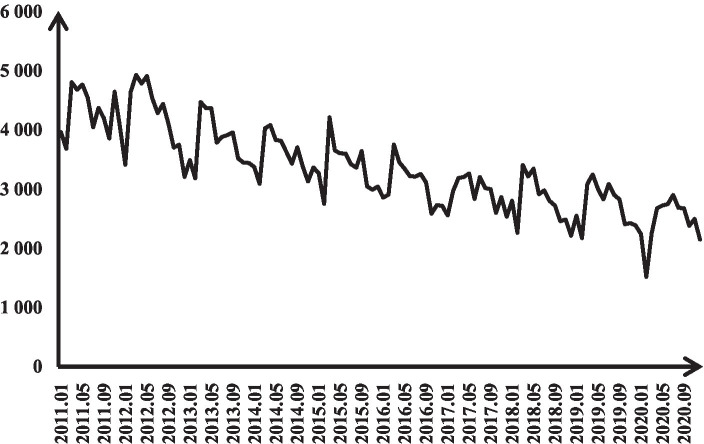
Tuberculosis prevalence in Jiangsu Province (2011–2020).

The cumulative number of reported cases of TB in Jiangsu Province from 2010 to 2020 was 399,508, with an annual average of 39,950 cases. Trend, seasonal and random error analysis of monthly incidence data from January 2010 to December 2019 (Figure 2) revealed a clear seasonality in the number of monthly TB cases. The epidemic peaks from March to July each year, followed by a declining trend in the number of cases, with random errors fluctuating within a certain range.

### Correlation analysis and time-series change characteristics

By calculating the correlation between the search terms and the actual morbidity data, the initial screening was carried out according to the |r| ≥ 0.5 and deleted the search terms with too low a frequency. In the end, 11 search terms with high correlation were initially screened. Its differences were statistically significant, and the search term correlation coefficients are shown in Table 1.

**Table 1:** Correlation coefficients for initial screening search terms.

Search term	*r*
Persistent low fever (持续低烧)	−0.53
Night sweats (盗汗)	−0.72
Cough (咳嗽)	−0.70
Sore throat (咽喉痛)	−0.55
Poor appetite (食欲不振)	−0.60
Early signs of tuberculosis (肺结核的早期症状)	−0.50
BCG vaccine (卡介苗)	−0.61
Difficulty in breathing (呼吸困难)	−0.59
Fatigue (乏力)	−0.59
Tuberculosis (肺结核)	−0.70
PPD (结核菌素试验)	−0.72

**Figure 3:**
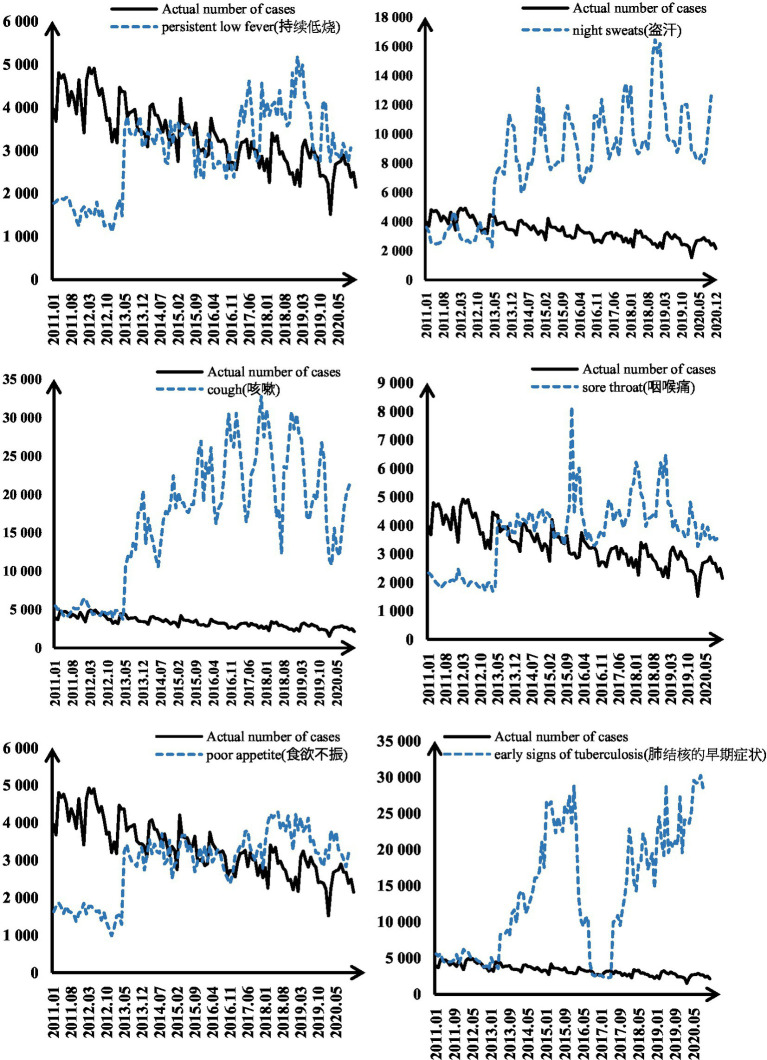
Trend between search term Baidu index and actual data.

Then the correlation coefficients of 11 search terms in “leading 2 months” (*d* = 2) were calculated and compared with the simultaneous ones. The differences were statistically significant, *p* < 0.05. As shown in Table 2, the six search terms with “leading” characteristics were screened. Figure 3 shows the trend between the “leading” search terms and the actual incidence data. Before 2015, the Chinese Internet was still in its infancy. In the same time, the Internet healthcare was still in its infancy. Medical treatment, medical information and disease knowledge science were the main themes at this stage. People were not yet familiar with using Baidu to search for knowledge related to tuberculosis. So the model missed a peak in 2015. After a period of a new pandemic, the frequency of search terms for “respiratory symptoms” increases rapidly until the beginning of 2020. The lag in reporting of case numbers. So the data show that the search terms exceed the beginning of COVID in 2020.

**Table 2:** Correlation coefficients for “2 months ahead” search terms.

Search term	Synchronization (*r*)	leading 2 months (*r*)
Persistent low fever (持续低烧)	−0.53	−0.58
Night sweats (盗汗)	−0.72	−0.79
Cough (咳嗽)	−0.70	−0.71
Sore throat (咽喉痛)	−0.55	−0.63
Poor appetite (食欲不振)	−0.60	−0.61
Early signs of tuberculosis (肺结核的早期症状)	−0.50	−0.56

### Multiple linear regression model

#### Modeling

There was a “2-month time” lag between the input and the output variables. The “leading” search term Baidu index in January was used to predict the prevalence and intensity of TB in March. The input variables were the Baidu index of “leading” search terms from January 2011 to October 2020, and each input variable was statistically different from the other (*p* < 0.05). The output variable was the monthly incidence prediction data from March 2011 to December 2022, of which the proportion of the training set is 90%. The independent variable is the Baidu index (x_1_, x_2_, x_3_, …, x_6_) of the leading search terms [“persistent low fever (持续低烧),” “night sweats (盗汗),” “cough (咳嗽),” “sore throat (咽喉痛),” “loss of appetite (食欲不振),” “early symptoms of tuberculosis (肺结核的早期症状)”]. The dependent variable is the actual incidence of tuberculosis (*y*). The regression model was obtained by selecting the “input” method for all the independent variables. According to the SPSS 26.0 output, a multiple linear regression model was obtained:


(4) $$\begin{array}{c} y=0.134x_{1}-0.163x_{2}-0.016x_{3}+0.156x_{4}\\ -0.103x_{5}-0.015x_{6}+4470.978 \end{array}$$

Finally, the results showed that *F* is 37.968 and the difference was statistically significant (*p* < 0.05), indicating a linear relationship between the independent and dependent variables.

#### Forecast results

According to the forecast results in Table 3, the relative error of the forecast for other months is mostly between 10 and 20%, which is relatively small and the forecast effect is relatively accurate. Considering the offset caused by the “Spring Festival effect,” there was large the relative error of the forecast prediction in March.

**Table 3:** Multiple linear regression model prediction results.

Date	Predicted value	Actual value	Relative error
2020.03	3,123	2,253	38.61%
2020.04	3,155	2,674	17.98%
2020.05	3,054	2,719	12.32%
2020.06	3,099	2,744	12.93%
2020.07	3,129	2,898	7.97%
2020.08	2,939	2,679	9.70%
2020.09	2,585	2,676	3.40%
2020.10	2,272	2,379	4.49%
2020.11	2,237	2,493	10.27%
2020.12	2,312	2,146	7.73%

#### Evaluation of the results

The degree of fit of the model was evaluated using the mean absolute value (*MAE*) and the mean absolute percentage error (*MAPE*) to evaluate the error of the model, which represents the mean of the absolute errors between the predicted values, and the smaller and the better the prediction (Table 4).

**Table 4:** Multiple linear model prediction errors.

Evaluation indicators	Multiple linear regression
*MAE*	318.06
*MAPE*	10.23%
*R^2^*	0.67

**Figure 4:**
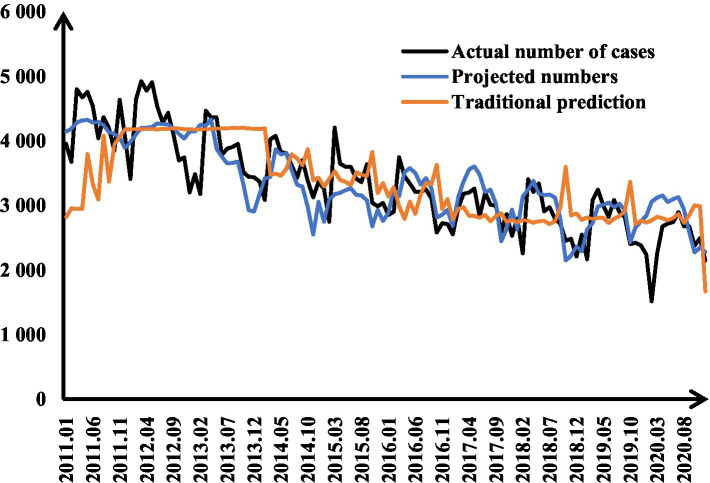
Visualization of predicted results.

Figure 4 shows the visualization of “leading 2-month” model predicted values. Firstly, in terms of the overall predictive value, the fit and predictive effect of the multiple linear regression model is satisfactory, with little difference between the predicted and actual values, and the goodness of fit test result is 0.672, which means the variable can explain 67.2% of the variation in the dependent variable. It indicates the predictive model has some extrapolation. At the same time, the predicted results were basically the same as the epidemic trend of the actual situation. The predicted emergence of the epidemic wave was basically consistent with the time point of the actual incidence. The multiple linear regression has good predictive ability and can predict the epidemic trend of tuberculosis in a timely and effective manner.

## Conclusion

### There is a linear relationship between the search term Baidu index and the actual morbidity data

In terms of search behavior, the search terms chosen for this study were consistent with the logic of search behavior. The search terms used in this study cover the four main categories of prevention, treatment, symptoms and common terms for tuberculosis, which can make full use of the health information of suspected infected and susceptible people before they go to the clinic. Bringing forward the predicted juncture to the incubation period or early onset. Secondly, the correlation analysis confirmed that there was a linear correlation between the Baidu index data and the actual data. Among them, 11 search terms were highly correlated with the actual incidence, indicating the potential effectiveness of the Baidu index in predicting the prevalence of tuberculosis. Among the search terms initially screened, those with “synchronous” and “lagging” characteristics were eliminated by calculating the time series change of correlation, by filtering the search terms with “leading” characteristics, the prediction point is further advanced to the pre-pandemic period.

### The forecast results are time-sensitive

Due to the “2-month time” lag between the input and output variables of the model, the TB prediction model developed in this study is able to predict the next wave of TB epidemic trends and intensity 2 months in advance, which is different from traditional prediction models. The traditional models were based on previous incidence data. It’s principle is to predict outcomes by analyzing patterns in historical data. The data source of this study is the Baidu index, which has the characteristics of real-time, rapid and large amount of internet search data. According to their own symptoms, the incubation period of tuberculosis and susceptible people generates health information search behavior, Then, according to the search behavior, the generation of Baidu index is real-time. It can effectively capture the dynamic changes of the real prevalence situation and monitor the infection and prevalence of tuberculosis in a timely manner. Therefore, the prediction model has a strong timeliness and can effectively capture the health information of latent and susceptible people and can predict the pandemic trend of TB in 2 months in advance.

## Discussion

In this paper, we construct a prediction model for infectious diseases using web search data, which is the same as the conclusion of other researchers. The search data can be a better complement to traditional surveillance data (17–23).

The innovation of this paper is the temporal correlation of search terms, which can predict the trend and intensity of the next wave of TB epidemic 2 months in advance. This is different from the findings of other researchers, where existing search terms are analyzed only at the level of correlation size without further exploration (24–29). In contrast, this paper provides an in-depth analysis of the time-series variation characteristics of search terms.

## Limitations

### Further screening of search terms with high specificity

The next step in the study is to identify search terms with high specificity. In this paper, the search terms “how to treat tuberculosis” and “tuberculosis treatment drugs” were selected mainly because people tend to search for more practical and cost-effective treatments on the Internet，which based on their search habits and disease progression. The search terms selected in this study were only classified from four aspects: “prevention,” “treatment,” “symptoms” and “commonly used words,” without considering other search terms. The search terms in this study mainly included pre-visit information of medical records. Solutions are sought online after the onset of some symptoms in the early stages of the disease. The specialized terms such as “BCG,” “chest x-ray “can only be learned after the consultation, and patients will follow the medical advice after the consultation rather than searching online. Therefore, the terminology of clinical diagnosis was not included in this study. It is not comprehensive enough and may lead to the omission of some search terms with high specificity. The next study should take the non-linear relationship into account, analyzing the relationship between the search terms and the actual data. In addition, eliminate the redundant data and retain the data with high specificity.

### Extrapolation of the Baidu index-based prediction model to predictions related to respiratory diseases

The search terms selected for this study included common symptoms of respiratory infectious diseases. But, this study only explored the relationship between the search terms and the incidence data of tuberculosis, without further extending to other respiratory diseases. Future studies should not only focus on the specificity of the search terms, but also should take the universality into account.

The results and findings of this study could be assessed for other respiratory diseases. To capture and detect trends in the prevalence of infectious diseases in a timely manner, and predict the peak of outbreaks in advance to minimize the impact of disease transmission on patients’ lives and property.

### Baidu index predictions should be extrapolated to other parts of China

Due to the differences in Baidu indexes between different provinces in China, the findings of this paper show that the forecasting method is feasible only in Jiangsu Province. Further studies should extend the model from this study to other areas of China.

## Data availability statement

The raw data supporting the conclusions of this article will be made available by the authors, without undue reservation.

## Author contributions

YW: research topic selection, design, data processing and analysis, and writing thesis. HZ: data checking and analysis and revising the thesis. ML: research supervision, statistical analysis of data, and participation in data analysis and interpretation. LZ: research supervision, statistical analysis of data, and participation in data analysis and interpretation. BH: research idea development and research process coordination. All authors contributed to the article and approved the submitted version.

## Funding

This work was supported by the Science and Technology Program Project of Xuzhou (No. KC20200) and Postgraduate Research and Practice Innovation Program of Jiangsu Province (No. SJCX23_1407).

## Conflict of interest

The authors declare that the research was conducted in the absence of any commercial or financial relationships that could be construed as a potential conflict of interest.

## Publisher’s note

All claims expressed in this article are solely those of the authors and do not necessarily represent those of their affiliated organizations, or those of the publisher, the editors and the reviewers. Any product that may be evaluated in this article, or claim that may be made by its manufacturer, is not guaranteed or endorsed by the publisher.
